# O20 A case series of paediatric primary Sjögren’s syndrome: differential diagnoses and multidisciplinary management

**DOI:** 10.1093/rap/rkaa054.008

**Published:** 2020-11-03

**Authors:** Bella Dave, Smithson Gayle, Veena Patel, Jodie Montgomery-Cranny, Alan Mighell

**Affiliations:** r1 Leeds Dental Institute, Leeds, United Kingdom; r2 The Mid-Yorkshire Hospitals NHS Trust, Dewsbury, United Kingdom

## Abstract

**Case report - Introduction:**

Two cases of primary Sjögren’s syndrome presenting in a child with recurrent salivary gland enlargement are described. As a condition which rarely presents in childhood, these cases underline the need to consider more uncommon differentials in a child presenting with recurrent salivary gland swellings. They also highlight how well co-ordinated multidisciplinary care involving medical and dental specialist teams can achieve the best outcomes for patients.

**Case report - Case description:**

*Case 1:* An 8-year-old girl presented to Paediatric Rheumatology with non-mealtime related episodes of bilateral parotid pain and swelling and intermittent dry mouth. Dry eyes and joint pain were not reported, and she was systemically well. Anti-Ro, anti-La and rheumatoid factor were positive. Salivary gland ultrasound showed evidence of sialadenitis. Sjögren’s Syndrome was diagnosed and treatment with hydroxychloroquine and azathioprine reduced the swelling frequency and severity. Regular optician reviews were arranged, and she was also noted to have ‘soft teeth’. Review by Paediatric Dentistry revealed her dry mouth had been managed with acidic sweets, leading to erosive tooth surface loss of the primary dentition. Onward referral was made to Oral Medicine and a differential diagnosis of chronic parotitis of childhood was considered. Ultrasound review by Oral and Maxillofacial Radiology confirmed Sjögren’s Syndrome. Yearly ultrasound scanning was arranged to assess for disease progression and lymphoma development.

*Case 2:* A 17-year-old girl was referred to Oral Medicine with a diagnosis of Sjögren’s syndrome. She initially presented to Rheumatology 3 years previously with dry mouth, dry eyes, and intermittent bilateral swellings of the parotid and submandibular glands. The salivary gland swellings were associated with pain on eating and fatigue and improved with short courses of oral corticosteroids. Titres of anti-Ro60/52 antibodies were high, and a right parotid ultrasound guided biopsy showed a focus score of greater than 2 with changes consistent with Sjögren’s Syndrome. Hydroxychloroquine was initiated and the frequency and severity of the swellings improved. Referrals were made to Ophthalmology for a dry eye’s ocular assessment and retinal screening in view of hydroxychloroquine therapy. She regularly attended her dentist and managed her dry mouth with sugar free sweets. On review with Oral Medicine various non-acidic oral lubricants were recommended in addition to yearly ultrasound scanning.

**Case report - Discussion:**

Sjögren’s syndrome is a multisystem autoimmune disease characterised by focal lymphocytic inflammatory infiltrates within the exocrine glands, predominantly the salivary and lacrimal glands. It is rarely diagnosed in children but should be considered when there is recurrent swelling of the salivary glands. There is a higher incidence of recurrent parotitis, but ocular and oral symptoms are less common in children. Therefore, the usual diagnostic criteria have a lower sensitivity for Sjögren’s syndrome in children compared with adults.

The main differential was chronic parotitis of childhood; a poorly defined condition characterised by recurrent parotid inflammation that is generally associated with non-obstructive sialadenitis. It can present with recurrent, self-limiting episodes clinically resembling mumps and has a spectrum of symptoms varying from mild to incapacitating. There can be overlapping features between the two conditions, including similar ultrasonography changes and punctate sialectasis on sialography. However, most cases of chronic parotitis fully resolve over time without any long-term adverse impact, whereas Sjögren’s Syndrome is typically progressive.

The risk of developing non-Hodgkin’s lymphoma in Sjögren’s is higher than the normal population. Therefore, patients should regularly undergo ultrasound surveillance, for example, by an Oral and Maxillofacial Radiologist. Immunosuppressant therapy may control symptoms and reduce lymphocytic infiltration and fibrosis of the salivary glands. However, there are no studies on systemic therapy for juvenile Sjögren’s and therefore management is based on that for adults. Hydroxychloroquine is widely used for reducing symptoms of fatigue and joint pain, whereas azathioprine is usually reserved for cases with systemic complications, such as lung disease. More recent studies evaluating the use of Rituximab, an anti-CD20 antibody have given mixed results, although it potentially could be considered in younger patients as long-term treatment with hydroxychloroquine or azathioprine carries an increased risk of side-effects.

**Case report - Key learning points:**

Several medical and dental specialities were involved in the management of these unusual cases of Sjögren’s Syndrome presenting in childhood and adolescence to deliver high quality, coordinated care. This underlines the importance of a multidisciplinary approach to deliver holistic young adult/child-centred care. Multidisciplinary management of Sjögren’s syndrome should include a General Paediatrician, a Paediatric or general medical Rheumatologist, an Ophthalmologist and Oral Medicine specialist. Involvement of the General Dental Practitioner is also recommended to maintain a functional dentition from an early age.

Although rare, Sjögren’s syndrome and recurrent parotitis of childhood should always be considered as differentials in a child with recurrent salivary gland swelling. A lower incidence of sicca complex symptoms can lead to difficulties in diagnosis; therefore, it is probable that Primary Sjögren’s syndrome is underdiagnosed in childhood. Regular ultrasound surveillance should be performed to monitor for non-Hodgkin’s lymphoma.

Treatment is largely symptomatic; both patients were managed with hydroxychloroquine and the first was also taking azathioprine. Potentially treatment may continue for several years. Long-term use of hydroxychloroquine carries a dose-dependent risk of retinopathy and requires regular optician and ophthalmology reviews. Long term use of azathioprine carries potential risks of lymphoma, non-melanoma skin cancers and bone marrow suppression. Therefore, careful consideration needs to be given to the consent process to ensure there is shared decision making between the prescribing clinician, patient, and parent.

