# Correction to: O20 A case series of paediatric primary Sjögren’s syndrome: differential diagnoses and multidisciplinary management

**DOI:** 10.1093/rap/rkac044

**Published:** 2022-06-02

This is a correction to: Bella Dave, Gayle Smithson, Veena Patel, Jodie Montgomery-Cranny, Alan Mighell, O20 A case series of paediatric primary Sjögren’s syndrome: differential diagnoses and multidisciplinary management, *Rheumatology Advances in Practice*, Volume 4, Issue Supplement_1, October 2020, rkaa054.008, https://doi.org/10.1093/rap/rkaa054.008

In the originally published version of this manuscript, the second presenter’s name was incorrect. The second presenter’s name should read “Gayle Smithson” instead of “Smithson Gayle”.

These details have been corrected only in this correction notice to preserve the published version of record.

